# Toxic Epidermal Necrolysis Post COVID-19 Vaccination - First Reported Case

**DOI:** 10.7759/cureus.17215

**Published:** 2021-08-16

**Authors:** Mohamad Bakir, Hanan Almeshal, Rifah Alturki, Sulaiman Obaid, Areej Almazroo

**Affiliations:** 1 Department of Medicine and Surgery, College of Medicine, Alfaisal University, Riyadh, SAU; 2 Department of Dermatology, Prince Mohammed Bin Abdulaziz Hospital, Riyadh, SAU

**Keywords:** toxic epidermal necrolysis, vaccination, covid-19, stevens-johnson-syndrome, etanercept, case report.

## Abstract

Stevens-Johnson syndrome/toxic epidermal necrolysis (SJS/TEN) is a spectrum of acute, delayed-type hypersensitivity reactions that affect the skin and the mucous membranes. Medications are the culprit cause of these disorders in addition to infections and in very rare instances vaccinations. We report a case of TEN in a 49-year-old woman with no previous medical history. The disorder developed one week after receiving the first dose of COVID-19 vaccine with no other identifiable causes. The patient received two doses of tumor necrosis factor-alpha inhibitor (etanercept) and she stopped developing new lesions after two days of the initial dose; complete healing was observed after 22 days and no side effects were observed in our patient. This case demonstrates an extremely rare complication to the COVID-19 vaccine. The benefits of receiving the COVID-19 outweigh the potential risk.

## Introduction

Toxic epidermal necrolysis (TEN) is a rare immune-mediated, life-threatening skin reaction characterized by blistering and extensive epidermal detachment of more than 30% of body surface area. The incidence is estimated to be 0.4 to 1.9 cases per million population per year worldwide and an estimated mortality rate of 25% to 35% [1, 2]. Medication is usually the cause of TEN (e.g., certain antibiotics and antiepileptics) [3]. Vaccination-induced Stevens-Johnson syndrome (SJS)/TEN is rare, with less than twenty reported cases in the published literature, with the measles vaccine being reported to cause both SJS and TEN, varicella, smallpox, anthrax, tetanus, and influenza vaccines were reported to cause SJS alone, and MMR (measles, mumps, rubella), hantavirus and meningococcal B vaccines were reported to cause TEN [4, 5, 6]. The patient usually develops a fever and other flu-like symptoms one to three weeks after being exposed to medication followed by painful erythematous to purpuric skin lesions that tend to coalescence. Next erosions and vesiculobullous lesions and epidermal detachment over wide body surface area develop. Mucous membranes are also involved, and the patient develops oral ulcers, vaginal ulcers, and possible acute conjunctivitis [7]. In this paper, we report a case of TEN following the administration of the Pfizer COVID-19 vaccine (Pfizer, Inc., New York, USA).

## Case presentation

**Figure 1:**
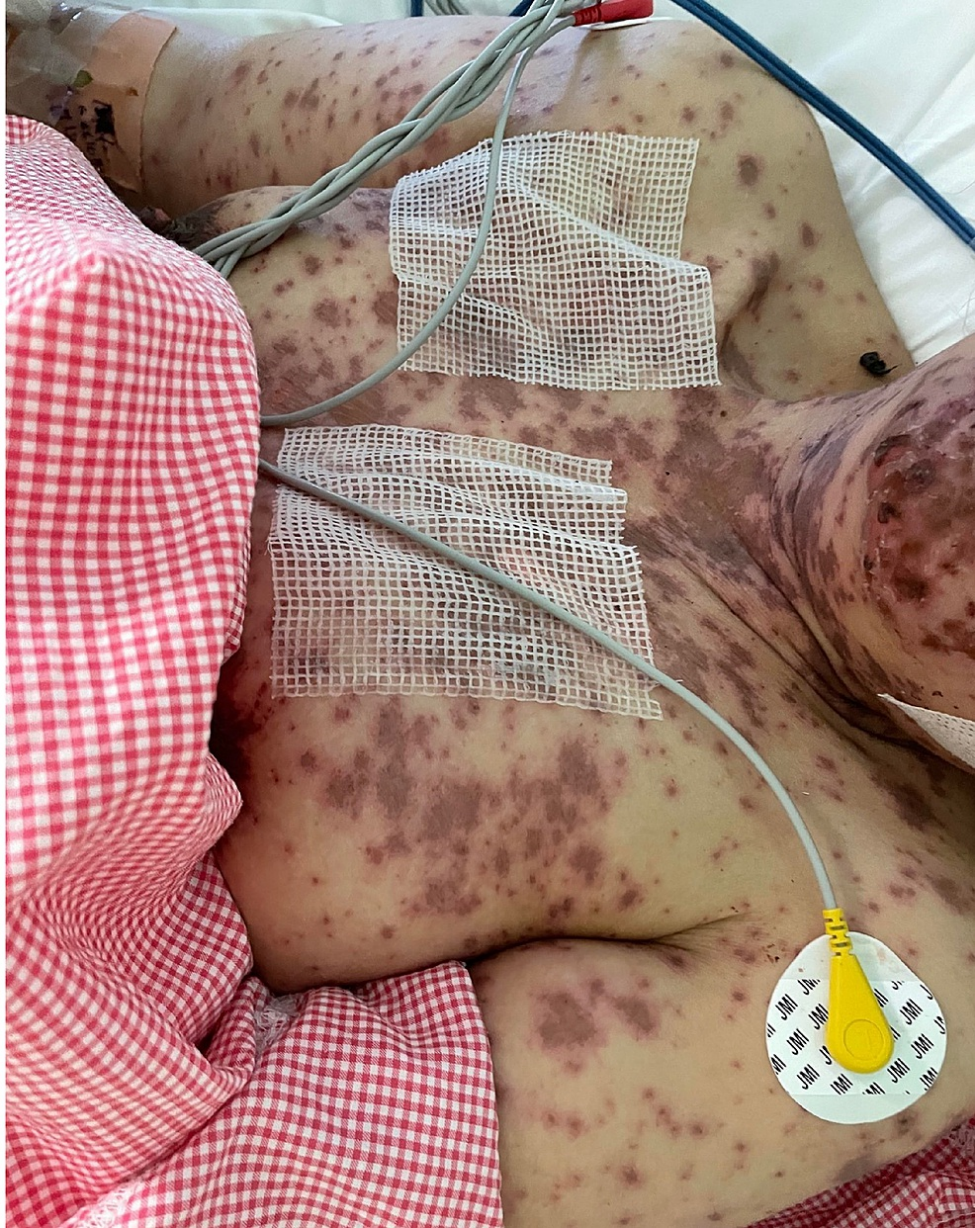
Overall image of the chest before initiating the treatment The image shows multiple purpuric patches with epidermal detachment affecting the chest.

**Figure 2:**
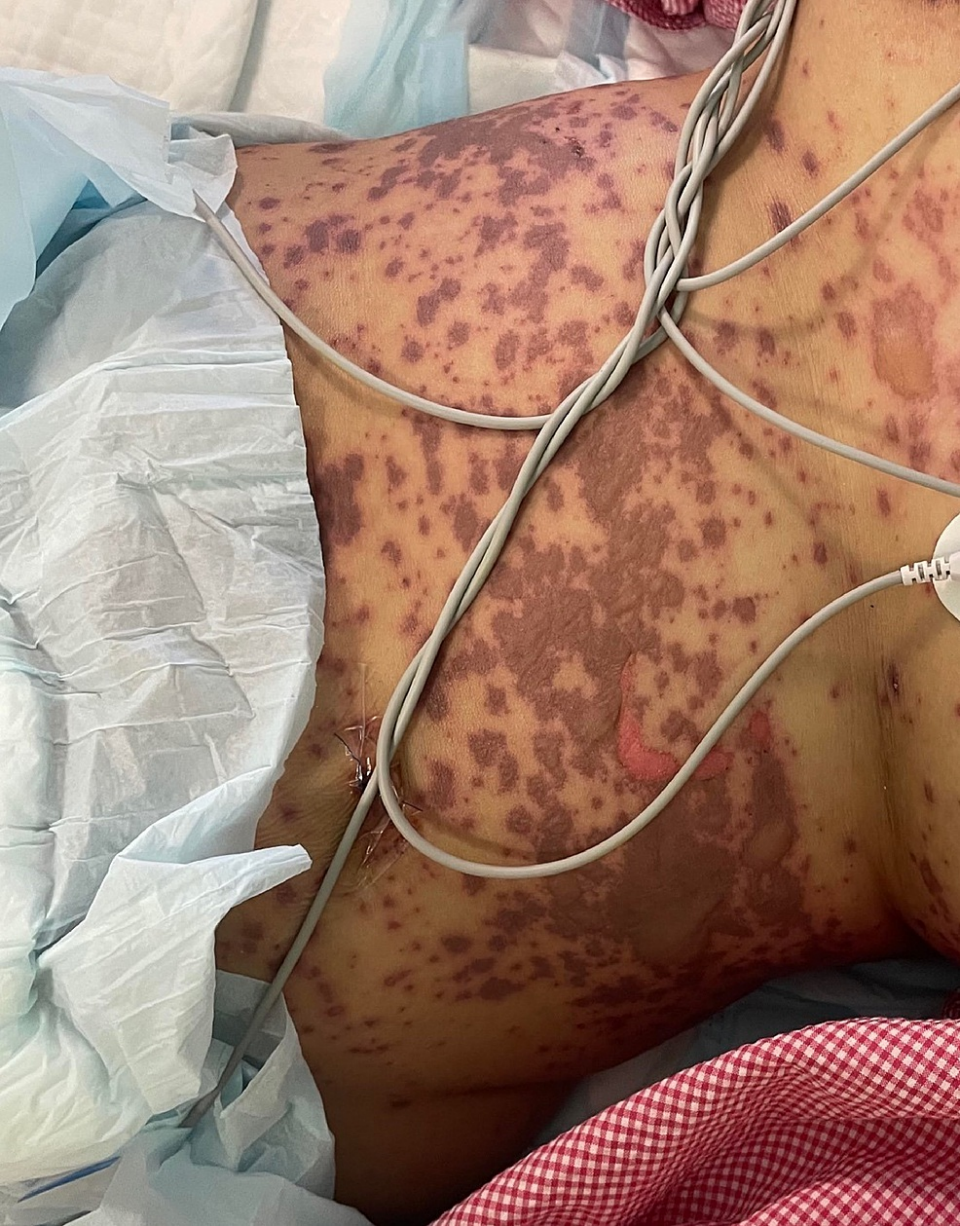
Overall image of the abdomen before initiating the treatment The image shows multiple purpuric patches with epidermal detachment affecting the abdomen.

**Figure 3:**
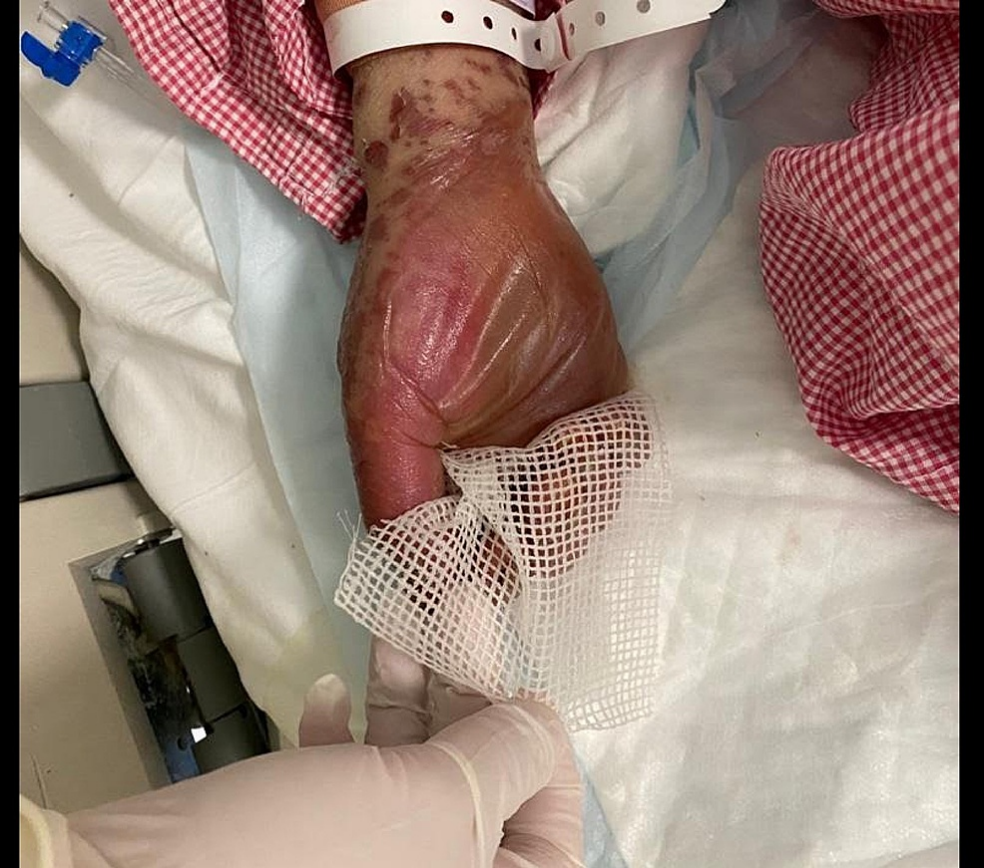
Overall image of the hand before initiating the treatment The image shows bullae and epidermal detachment affecting the palms.

**Figure 4:**
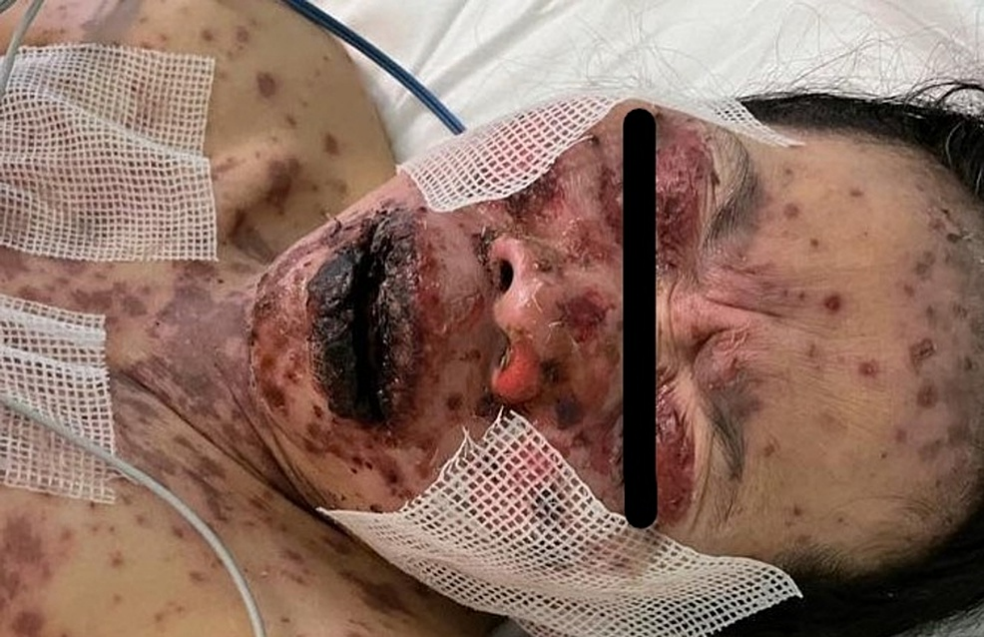
Overall image of the face before initiating the treatment The image shows severe hemorrhagic crusting of the lips.

**Figure 5:**
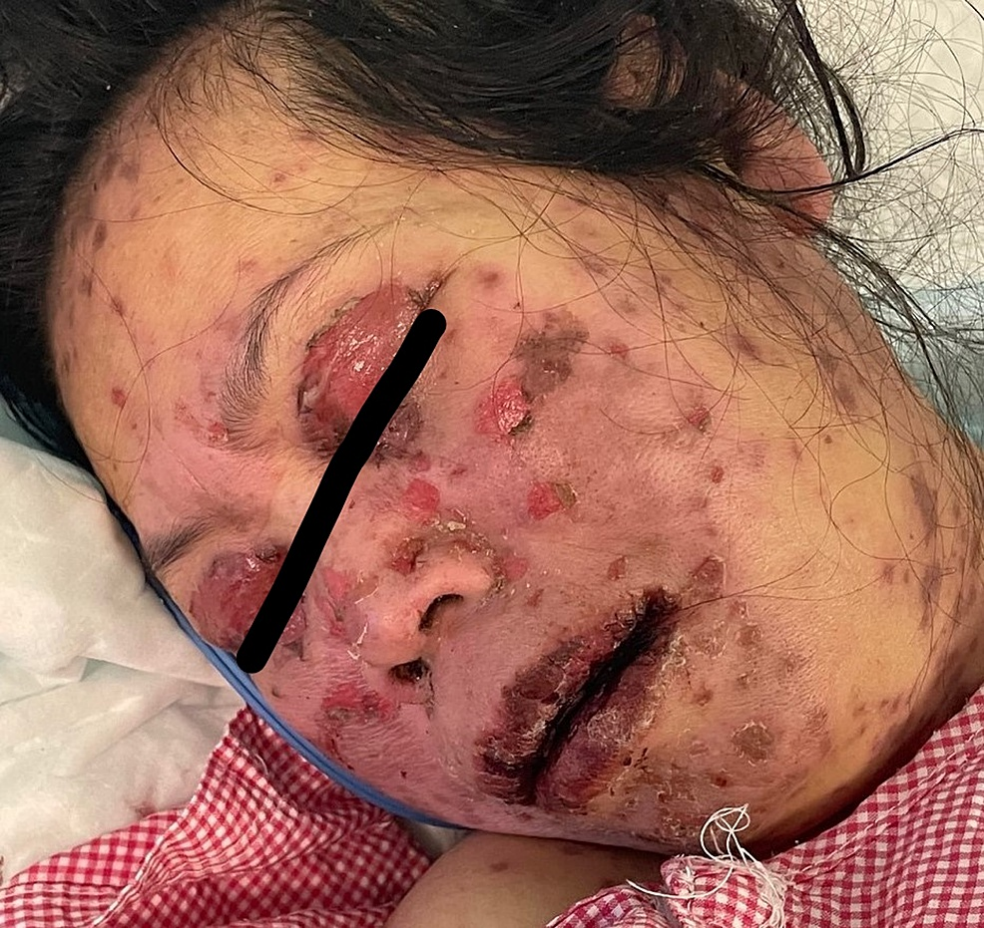
Overall image of the face before initiating treatment The image shows multiple erosions affecting the face and the upper eyelids.

A 49-year-old woman with no previous medical history presented to the emergency room with a history of fever and skin eruption. She has received COVID-19 Pfizer (BNT162b1) vaccine with a dose of 0.3 mL given intramuscularly one week before the development of her symptoms. The patient started to develop fever, fatigue, and headache followed by skin lesions affecting her trunk and starting to spread to her face and upper limbs with oral ulceration. The patient was seen in the primary health care center and was given paracetamol and did not notice any improvement. The patient had no history of taking any new medication or any cosmetic treatment in the past two months before the development of the skin lesions. Upon examination, the patient was vitally stable, anxious, and in severe pain. She had numerous purpuric and dusky red macules involving the chest (Figure 1), abdomen (Figure 2), upper limbs (Figure 3), face, genitalia, and upper thighs with areas showing coalescence of lesions with flaccid bullae and areas of epidermal detachment with positive Nikolsky's sign. The mucosa was involved in her condition, where she had extensive oral ulceration and hemorrhagic crusting over the lips (Figure 4), as well as bilateral conjunctival congestions along with upper eyelids erosions (Figure 5) and genital mucosal lesions. Her body surface area (BSA) involvement is estimated to be more than 30%. Laboratory evaluation showed low WBC (3.87 × 109/L) and elevated liver enzymes (aspartate aminotransferase [AST] 178 U/L, alanine aminotransferase [ALT] 90 U/L). Chest X-ray was normal, and she had negative serology for hepatitis B, C, and HIV. The Severity-of-Illness Score for Toxic Epidermal Necrolysis (SCORTEN) score was two on the day of her admission since she was older than 40 and she had a serum bicarbonate level of less than <20 mmol/L.

**Figure 6:**
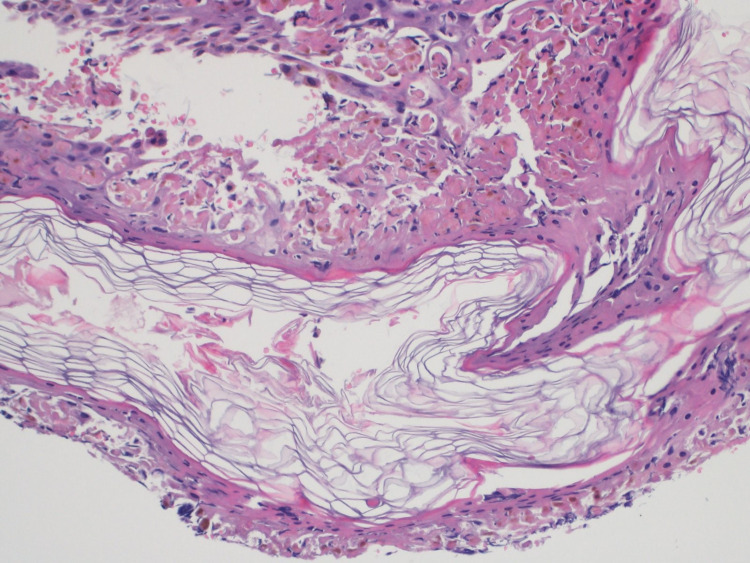
Biopsy before initiating the treatment The biopsy showed full-thickness epidermal necrosis along with dermal-epidermal separation and necrotic keratinocytes.

Histopathological examination of the lesion showed full-thickness epidermal necrosis along with dermal-epidermal separation and necrotic keratinocytes (Figure 6).

**Figure 7:**
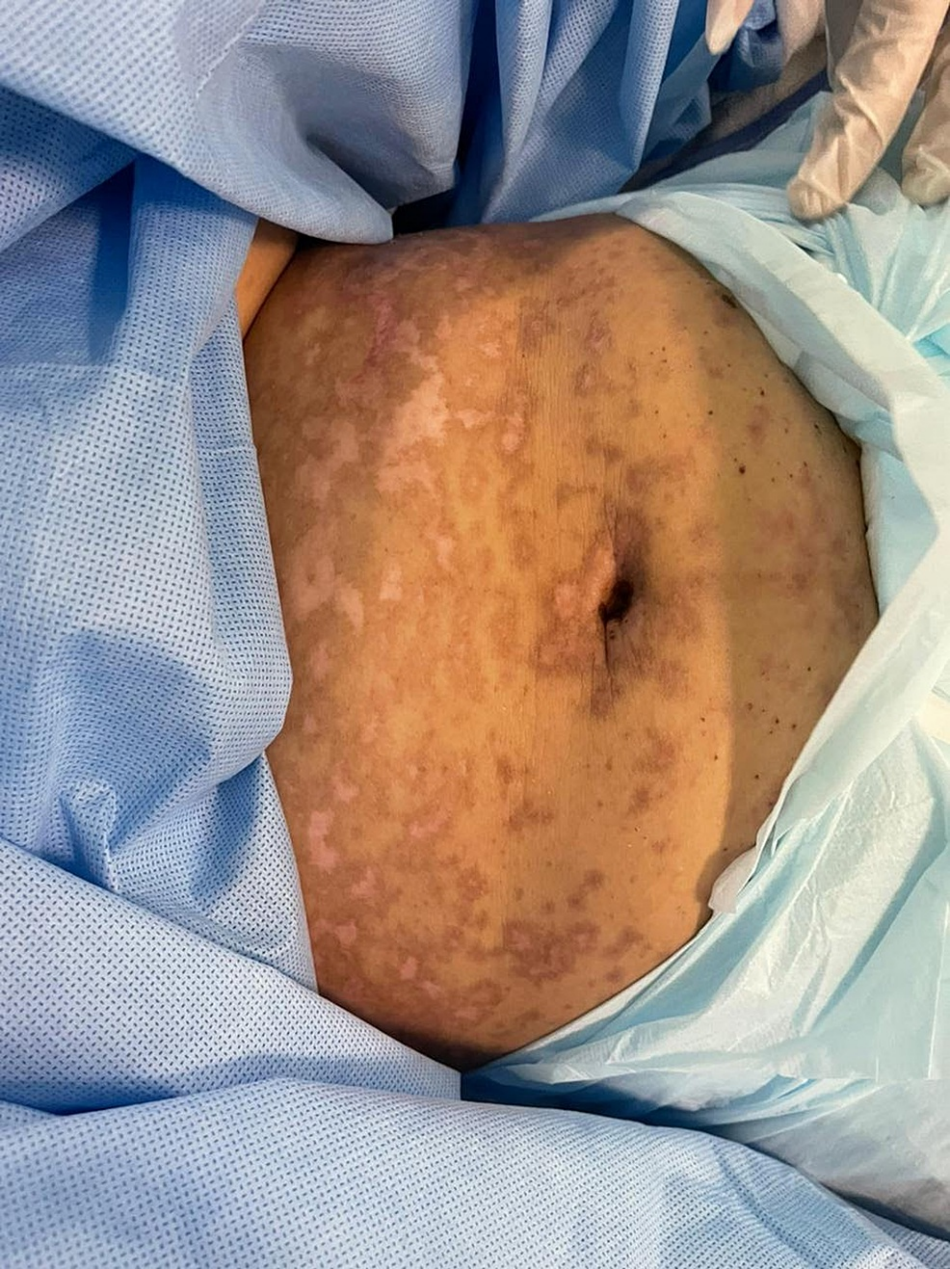
Overall image of the abdomen after the therapy The image shows areas of hyper-hypopigmentation involving the abdomen and chest.

**Figure 8:**
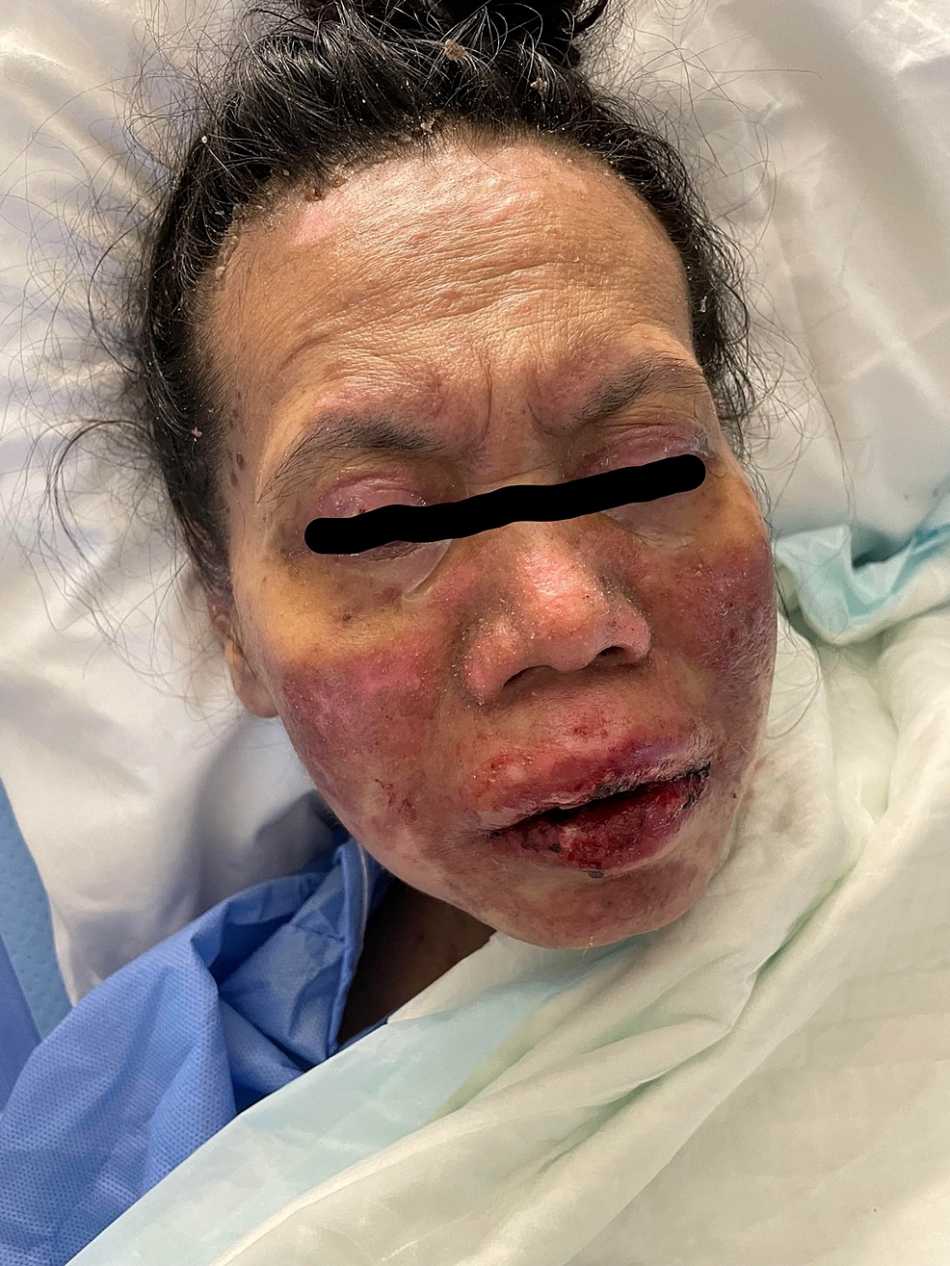
Overall image of the face after the therapy The image shows areas of hyper-hypopigmentation affecting the face.

The patient was admitted to the high dependency unit (HDU) and handled in an aseptic manner; IV fluid replacement and nutritional support were started. Ophthalmology, OB/GYN, and urology teams were involved in managing the case since her disease involved the eyes, and she needed topical antibiotics and topical lubricants. In addition, she had erosions that involved her genitalia so, topical vaseline gauze was applied and OB/GYN and urology teams followed her up to prevent future complications. The patient was given two doses of etanercept 50 mg/ml subcutaneously, the first one on the day of admission and the second one after two days of her admission. The patient stopped developing new lesions after two days from the first dose, and complete healing was noted after 22 days (Figures 7, 8). No side effects were noted in our patient.

## Discussion

**Figure 9:**
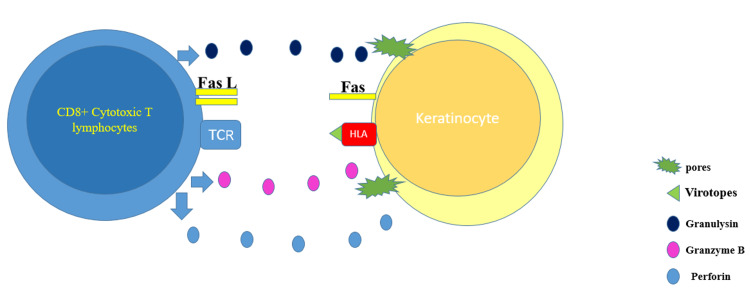
The graph depicts the pathogenesis behind virotopes antigen in the COVID-19 vaccine The graph shows how virotopes antigen in the COVID-19 vaccine can induce keratinocyte death by the activation of T lymphocytes. TCR: T cell receptor; HLA: human leukocyte antigen. The graph was created by Dr. Bakir using PowerPoint (Microsoft Corporation, Redmond, USA).

SJS/TEN represents a group of rare, severe, and potentially fatal delayed-type four-hypersensitivity reactions. Drugs are by far the most common cause of these conditions. However, SJS/TEN could be in very rare instances caused by vaccination [5]. The presentation starts with flu-like symptoms, followed by a painful rash that spreads and blisters. TEN lesions consist of targetoid lesions and purpuric macules with full-thickness epidermal necrosis, along with mucous membrane involvement. TEN is considered a medical emergency that requires urgent medical intervention, whereby the offending agent if present should be discontinued immediately. The most involved sites in SJS/TEN are the face, palms, soles, and the presternal area of the torso. In addition, the genital, ocular, and buccal mucosa are commonly involved [8]. In SJS, less than 10% of the body surface area is involved, whereas in TEN, more than 30% of the body surface area is involved. If the skin involvement is between 10% and 30%, it is considered as SJS-TEN overlap. The pathophysiology behind the development of SJS/TEN is believed to be drug-specific CD8+ lymphocytes, where these cytotoxic T cells release granulysin, granzyme B, and perforin - these enzymes lead to pores formation and activation of death cascade in the affected cells. In addition, tumor necrosis factor (TNF)-alpha and interferon-gamma are secreted in significant amounts by activated T cells, leading to Fas ligands overexpression and Fas-mediated keratinocyte death [9] (Figure 9).

COVID-19 vaccine is made of two components (excipients and virotopes). It has been hypothesized that the virotopes antigen, which is expressed on the keratinocyte surface, might lead to cytotoxic T lymphocyte activation and epidermal cells death [10, 11]. SJS/TEN is a clinical and histopathological diagnosis. On histopathology, there is necrosis of keratinocytes and dermal lymphocytic infiltrate with negative target immunofluorescent test. Patients with TEN require supportive care such as discontinuation of the offending agent(s), intensive care or burn unit admission, fluid and electrolyte replacement, nasogastric tube feeding, or total parenteral nutrition along with pain and temperature control. Sepsis and organ failure are the most feared complication of TEN. TNF-alpha levels were shown to be elevated in skin biopsy specimens, blister fluid, and serum of TEN patients, encouraging the use of biologic therapy that utilizes anti-TNF-alpha action [12, 13]. In one of the studies, a single dose of etanercept (Enbrel, Immunex Corporation, Seattle, USA) 50 mg/mL subcutaneously seemed to modify the course of the disease where significant reduction of edema and stop of disease development was noticed in just 24 hours [14]. In recent years, TNF-alpha antagonists have been used, and the majority of cases exhibited significant improvement with lesion formation ceasing within two days and complete resolution occurring within 20 days. Also, in SJS/TEN patients treated with etanercept, no severe side effects have been documented. In addition, as compared to individuals who were given corticosteroids, there was a lower rate of gastrointestinal hemorrhage and decreased granulysin and TNF-alpha expression levels [15]. Plasmapheresis, intravenous immunoglobulin (IVIG), and perhaps most promisingly TNF inhibitors such as etanercept are all treatment possibilities for vaccine-induced TEN [14]. However, the use of IVIG and high-dose systemic corticosteroids is still controversial [16]. Plasmapheresis is believed to work in TEN patients based on the premise of removing the drug, drug metabolite, or cytotoxic mediator from the circulation. However, one Swedish study found no benefit with plasmapheresis in eight patients when compared to patients in other studies who received equivalent support care but not plasmapheresis [17]. At this stage, there is insufficient data to justify the use of plasmapheresis above other adjunctive methods [18]. In a randomized controlled trial comparing the efficacy of etanercept and corticosteroids in SJS/TEN patients, etanercept was found to promote skin and oral mucosa healing and facilitate re-epithelialization [15]. To our knowledge, this is the first reported case of TEN secondary to the Pfizer COVID-19 vaccine. There were two cases of SJS post-COVID-19 vaccine, in the first case, they did not mention the name or the dose of the vaccine that their patient had received [11]. However, in the second case, they mentioned that their patient developed SJS after he received the second dose of the Pfizer COVID-19 vaccine [19]. Our patient had a full recovery after receiving two doses of etanercept.

## Conclusions

This instance highlights an extremely rare vaccine consequence. But the benefits greatly outweigh the risks in the present circumstances, therefore there should be no hesitation among the community to seek vaccination. Therefore, as we report this case, we emphasize the rarity of the occurrence of this side effect, and given the circumstances, this should not influence the decision of taking the vaccine, nor add to the misconceptions out there. In our case, we explained the pathophysiology behind the development of toxic epidermal necrolysis secondary to vaccination and we highlighted the successfulness of eternacept as a safe and fast treatment of this condition.
